# An investigation of seasonal variations in the microbiota of milk, feces, bedding, and airborne dust

**DOI:** 10.5713/ajas.19.0506

**Published:** 2019-12-24

**Authors:** Thuong Thi Nguyen, Haoming Wu, Naoki Nishino

**Affiliations:** 1 Department of Animal Science, Graduate School of Environmental and Life Science, Okayama University, Okayama 700-8530, Japan

**Keywords:** Cowshed, Dairy Cow, Microbiota, Milk, Season

## Abstract

**Objective:**

The microbiota of dairy cow milk varies with the season, and this accounts in part for the seasonal variation in mastitis-causing bacteria and milk spoilage. The microbiota of the cowshed may be the most important factor because the teats of a dairy cow contact bedding material when the cow is resting. The objectives of the present study were to determine whether the microbiota of the milk and the cowshed vary between seasons, and to elucidate the relationship between the microbiota.

**Methods:**

We used 16S rRNA gene amplicon sequencing to investigate the microbiota of milk, feces, bedding, and airborne dust collected at a dairy farm during summer and winter.

**Results:**

The seasonal differences in the milk yield and milk composition were marginal. The fecal microbiota was stable across the two seasons. Many bacterial taxa of the bedding and airborne dust microbiota exhibited distinctive seasonal variation. In the milk microbiota, the abundances of Staphylococcaceae, Bacillaceae, Streptococcaceae, Microbacteriaceae, and Micrococcaceae were affected by the seasons; however, only Micrococcaceae had the same seasonal variation pattern as the bedding and airborne dust microbiota. Nevertheless, canonical analysis of principle coordinates revealed a distinctive group comprising the milk, bedding, and airborne dust microbiota.

**Conclusion:**

Although the milk microbiota is related to the bedding and airborne dust microbiota, the relationship may not account for the seasonal variation in the milk microbiota. Some major bacterial families stably found in the bedding and airborne dust microbiota, e.g., Staphylococcaceae, Moraxellaceae, Ruminococcaceae, and Bacteroidaceae, may have greater influences than those that varied between seasons.

## INTRODUCTION

The assessment of milk microbiota is important for preventing mastitis and maintaining herd health [1–3]. If typical contagious bacteria, such as *Staphylococcus aureus* (*S. aureus*), *Streptococcus agalactiae* (*S. agalactiae*), and *Corynebacterium bovis* (*C. bovis*), are found in the tank milk, the infected cows should be identified, and the procedure and sequence of milking should be revised. If environmental bacteria, such as coliforms, coagulase-negative *Staphylococci*, and *Streptococci* other than *S. agalactiae*, markedly increase, the hygiene of the cowshed should be improved. Likewise, pathogen identification greatly facilitates the provision of appropriate treatment with antibiotics. Regarding the quality control of milk, the abundance of *Pseudomonas* spp. may be of great concern, because their heat-resistant enzymes can hydrolyze protein and fat, producing an unpleasant flavor even after pasteurization [4,5]. Moreover, several *Pseudomonas* spp. cause mastitis [6].

The prevalence of mastitis varies according to the season in dairy cows as does the somatic cell count (SCC), which is an indicator of the number of leukocytes in the milk and therefore of udder health. Makovec and Ruegg [7] reported that mastitis caused by *Escherichia coli* (*E. coli*) and *Streptococcus* spp. may occur more during summer, and Olde Riekerink et al [2] described the greater risk of mastitis caused by *S. aureus* and *Streptococcus dysgalactiae* during winter. Olde Riekerink et al [2] also reported the effect of housing on seasonal variation; mastitis caused by *E. coli* can occur more during summer if cows are managed on pasture, whereas the infection may be seen more during winter if managed as confined herds. Mastitis caused by coagulase-negative *S. aureus* and *C. bovis* may occur throughout the year. Milk spoilage caused by psychrophilic *Pseudomonas* spp. can become a problem during winter.

Regardless of the symptoms of mastitis, the milk microbiota varies between seasons [8]. Differences in temperature and humidity could account for the variation, because both the health of the cows and the growth of milk bacteria are influenced by temperature and humidity. However, Li et al [9] found that at the phylum level the milk microbiota during summer was similar to that during winter. In fact, it is unclear what causes seasonal variations in the milk microbiota.

Among the factors putatively involved in the milk microbiota and mastitis outbreak, the bedding microbiota may be the most important because the teats of a dairy cow are in direct contact with bedding material when the cow is resting. In a previous study, we examined the microbiota of the gut, milk, and cowshed environment using 16S rRNA gene amplicon sequencing, and demonstrated that the milk microbiota is associated with the bedding microbiota but is clearly distinct from the feed, rumen fluid, fecal, and water microbiota [10]. We conducted the survey at two farms: one in April and one in September; hence, the difference in the milk microbiota between the farms might have resulted from seasonal variations. Although the importance of the bedding microbiota has long been recognized from the perspective of mastitis prevention, few studies have examined the milk and cowshed microbiota or the variation across seasons.

In the present study, we collected samples of milk, feces, bedding, and airborne dust from a dairy farm during summer and winter at 1 and 2 months postpartum. The microbiota was assessed by 16S rRNA gene amplicon sequencing. The objective was to determine if the milk and cowshed microbiota vary between seasons, and if a variation in the milk microbiota is related to a variation in the cowshed microbiota.

## MATERIALS AND METHODS

### Sampling

We collected samples from cows at the Okayama Prefecture Livestock Research Institute (Okayama, Japan). The cows were housed in a free stall barn and fed a total mixed ration silage throughout the year (Table 1). During the summer their diet was supplemented with a fatty acid salt (palmitic acid calcium) to fortify milk fat production. The contents of dry matter (DM), crude protein (N×6.25), and total digestible nutrients were 55% to 60%, 16% to 17%, and 72% to 4% DM, respectively. The sampling was performed from 6 June to 22 August and from 17 November to 16 January. Hereafter, the former series is referred to as the summer sampling and the latter as the winter sampling. The minimum and maximum temperatures were 16°C to 31°C during the summer and −2°C to 15°C during the winter.

The milk and feces samples were collected from 9 cows during summer and from 8 cows during winter, with 2 sampling times each at 1 and 2 months postpartum. Because milk and fecal sampling from cows at 1 and 2 months postpartum was occasionally conducted on the same day, bedding and airborne dust sampling was carried out 6 times during summer and 9 times during winter.

Each milk sample was collected after cleaning the surface of the udder, and the foremilk was discarded before collecting the sample. Milk samples were taken manually from 4 udders, then mixed to produce a composite sample. Fecal samples were collected from the rectum. Airborne dust samples were collected by placing 3 petri dishes approximately 1.0 m above the ground for 5 min. Bedding samples were collected from 3 separate places in a cowshed. In the free stall system, cows can move and rest freely, and determining their resting place was difficult. Thus, a composite sample prepared from 3 separate samples was regarded as a representative means of assessing the bedding and airborne dust microbiota at the time of sampling. The institute operated automatic milking systems (Lely Astronout A4, Cornes AG. Ltd., Eniwa, Japan) that enabled cows to be milked at any time; hence, although all samplings were completed between 10:00 and 12:00, the time when we collected the milk after milking was variable. All the samples were kept on ice during transportation to the laboratory and were stored at −20°C until required for further analyses. Overall, 34 milk, 34 feces, 15 bedding, and 15 airborne dust samples were subjected to MiSeq analysis.

The contents of protein, fat, and solids-not-fat (SNF), and the SCC of the milk were determined using a CombiFoss FT+ analyzer (Foss Allé, Hillerød, Denmark). The procedures and protocols for the animal experiments were approved by the Animal Care and Use Committee, Okayama University (OKU-2016290), Japan.

### DNA extraction

The 250 μL milk samples were centrifuged at 16,000 g for 2 min, and the pellet was collected. For DNA extraction from airborne samples, a 1-mL aliquot of each sample was transferred into an Eppendorf tube, and then centrifuged to collect the pellet. All the pellet samples were washed with 500 μL of solution I containing 0.05 M D-glucose, 0.025 M Tris-HCl (pH 8.0), and 0.01 M sodium ethylenediaminetetraacetic acid (EDTA) (pH 8.0), and then lysed with 180 μL of lysozyme solution (20 g/L lysozyme, 0.02 M Tris-HCl [pH 8.0], 0.002 M sodium EDTA [pH 8.0], 1.2 g/L Triton X-100) at 37°C for 1 h. Bacterial DNA of milk, airborne dust samples was purified by using the DNeasy blood & tissue kit (Qiagen, Germantown, MD, USA), according to the manufacturer’s instructions. For feces and bedding samples, a 0.2 g of the sample was used for bacterial DNA extraction following the procedure for the repeated bead beating plus column method [11] and purified using the mini DNeasy stool kit (Qiagen, USA).

### 16S rRNA gene amplicon sequencing

Bacterial DNA was amplified by two-step polymerase chain reaction (PCR) to generate amplicon libraries for next-generation sequencing [12]. The primers targeting the V4 region of 16S ribosomal RNA (rRNA) genes (forward: 5′-ACACTC TTTCCCTACACGACGCTCTTCCGATCTGTGCCAGCMGCCGCGGTAA-3′; reverse: 5′-GTGACTGGAGTTCA GACGTGTGCTCTTCCGATCTGGACTACHVGGGTW TCTAAT-3′) were used for the first round of PCR, with the following protocol: initial denaturation at 94°C for 2 min, followed by 35 cycles of denaturation at 94°C for 30 s, annealing at 50°C for 30 s, elongation at 72°C for 30 s, and an elongation step at 72°C for five min. The PCR products were purified by electrophoretic separation on a 2.0% agarose gel using a Fast Gene Gel/PCR Extraction Kit (NIPPON Genetics Co., LTD., Tokyo, Japan). The second round of PCR, with adapter-attached primers, followed the protocol of initial denaturation at 94°C for two min, 10 cycles of denaturation at 94°C for 30 s, annealing at 59°C for 30 s, elongation at 72°C for 30 s, and an elongation step at 72°C for five min. The second-round PCR products were purified in the same way as that in case of the first-round PCR products.

The purified amplicons were pair-end sequenced (2×250 bp) on an Illumina MiSeq platform at FASMAC Co., Ltd. (Kanagawa, Japan). Raw sequence data were analyzed using the Quantitative Insights Into Microbial Ecology (QIIME version 1.9.0). The 250-bp reads were truncated at any site receiving an average quality score under 20. Truncated reads that were shorter than 225 bp were discarded. In primer matching, sequences showing overlaps longer than 200 bp were assembled. The final reads obtained after pair-end joining were grouped into operational taxonomic units using a 97% similarity threshold. The sequence data were analyzed and categorized from the phylum to the family level using the default settings of the Ribosomal Database Project classifier.

### Statistical analysis

Data pertaining to the milk yields, milk composition, blood metabolite concentrations, and relative abundances of major bacterial families in the milk, feces, bedding, and airborne dust microbiota (where the proportion of the family in at least one sample was >1.0%) were analyzed by the non-parametric Mann–Whitney U test to examine the effects of seasons and months after calving. The microbiota data were also subjected to canonical analysis of principal coordinates (CAP) to define assignment and clustering that explained the variations in the microbiota. Discriminant vectors with a Pearson correlation >0.7 were considered significant. The non-parametric test was performed using JMP software (version 11; SAS Institute, Tokyo, Japan) and CAP was carried out using Primer version 7 with the Permanova+ add-on (Primer-E, Plymouth Marine Laboratory, Plymouth, UK).

## RESULTS

The milk yield of the cows was 35 to 39 kg, and there were no differences between the two seasons or between 1 and 2 months after calving (Table 2). The content of milk protein was greater, and the contents of fat and SNF were numerically higher during winter than during summer. The average SCC of the milk at 2 months postpartum during summer reached 429×10^3^ cells/mL, because one cow had a markedly high SCC (2.8×10^6^ cells/mL).

At the family level, the five most abundant taxa of the milk microbiota during summer were Staphylococcaceae (10.3%), Ruminococcaceae (9.7%), Aerococcaceae (7.7%), Lachnospiraceae (5.4%), and Corynebacteriaceae (4.3%), and those during winter were Ruminococcaceae (10.2%), Staphylococcaceae (7.5%), Lactobacillaceae (6.5%), Aerococcaceae (5.5%), and Lachnospiraceae (5.3%) (Table 1). There were seasonal variations in the relative abundances of Bacillaceae, Micrococcaceae, and Staphylococcaceae (summer > winter) and those of Streptococcaceae, and Microbacteriaceae (summer < winter). There were also variations in the relative abundances of Porphyromonadaceae (1 month > 2 months), and Turicibacteraceae and Tissierellaceae (1 month < 2 months) between samples taken 1 or 2 months postpartum.

**Figure 1:**
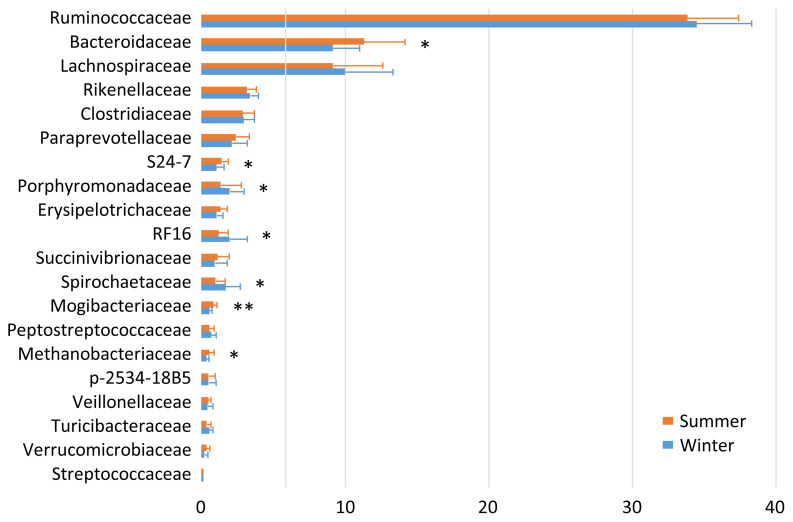
Family-level proportions of the top 20 bacterial taxa of the fecal microbiota of the dairy cows investigated during summer and winter. Bars indicate mean values with standard deviations. Asterisks indicate significant differences between summer and winter.

The fecal microbiota was fairly stable at 1 and 2 months postpartum. Therefore, the data are summed up to enable comparison between the two seasons (Figure 1). Ruminococcaceae (34.1%), Bacteroidaceae (10.2%), Lachnospiraceae (9.6%), Rikenellaceae (3.3%), and Clostridiaceae (2.9%) were the five most abundant taxa regardless of the season. There were seasonal variations in the relative abundances of S24-7, Mogibacteriaceae, and Methanobacteriaceae (summer > winter), and of Porphyromonadaceae, RF16, and Spirochaetaceae (summer < winter).

**Figure 2:**
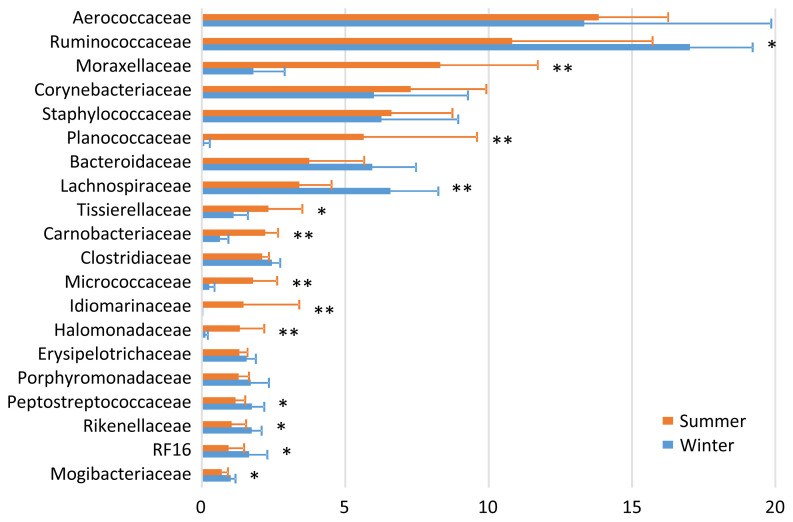
Family-level proportions of the top 20 bacterial taxa of the bedding microbiota of a dairy farm investigated during summer and winter. Bars indicate mean values with standard deviations. Asterisks indicate significant differences between summer and winter.

In the bedding microbiota, Aerococcaceae (13.8%), Ruminococcaceae (10.8%), Moraxellaceae (8.3%), Corynebacteriaceae (7.3%), and Staphylococcaceae (6.6%) were the five most abundant taxa during summer, and Ruminococcaceae (17.0%), Aerococcaceae (13.3%), Lachnospiraceae (6.6%), Staphylococcaceae (6.3%), and Corynebacteriaceae (6.0%) were the five most abundance taxa during winter (Figure 2). There were seasonal variations in the relative abundances of Moraxellaceae, Planococcaceae, Tissierellaceae, Carnobacteriaceae, Micrococcaceae, Idiomarinaceae, and Halomonadaceae (summer > winter); and of Ruminococcaceae, Lachnospiraceae, Peptostreptococcaceae, Rikenellaceae, RF16, and Mogibacteriaceae (summer < winter).

**Figure 3:**
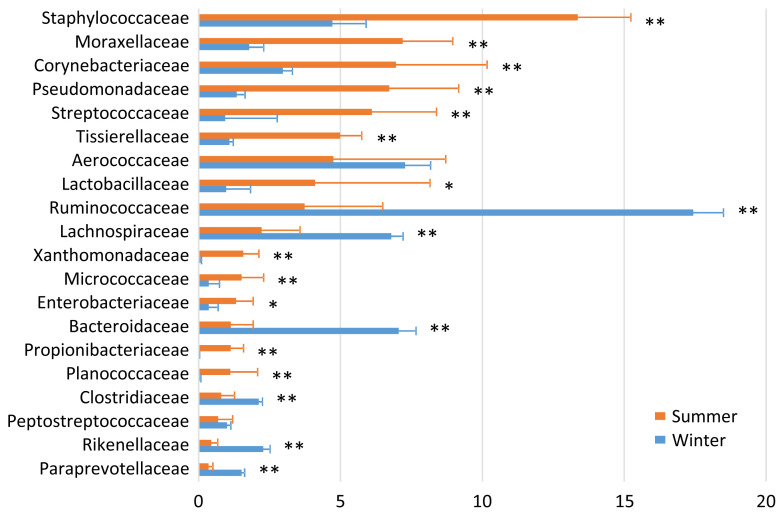
Family-level proportions of the top 20 bacterial taxa of the airborne dust microbiota of a dairy farm investigated during summer and winter. Bars indicate mean values with standard deviations. Asterisks indicate significant differences between summer and winter.

In the airborne dust microbiota, the five most abundant taxa during summer were Staphylococcaceae (13.4%), Moraxellaceae (7.2%), Corynebacteriaceae (7.0%), Pseudomonadaceae (6.7%), and Streptococcaceae (6.1%); and the five most abundant taxa during winter were Ruminococcaceae (17.4%), Aerococcaceae (7.3%), Bacteroidaceae (7.1%), Lachnospiraceae (6.8%), and Staphylococcaceae (4.7%) (Figure 3). There were seasonal variations in the relative abundances of Staphylococcaceae, Moraxellaceae, Corynebacteriaceae, Pseudomonadaceae, Streptococcaceae, Tissierellaceae, Lactobacillaceae, Xanthomonadaceae, Micrococcacae, Enterobacteriaceae, Propionibacteriaceae, and Planococcaceae (summer > winter); and of Ruminococcaceae, Lachnospiraceae, Bacteroidaceae, Clostridiaceae, Rikenellaceae, and Paraprevotellaceae (summer < winter).

**Figure 4:**
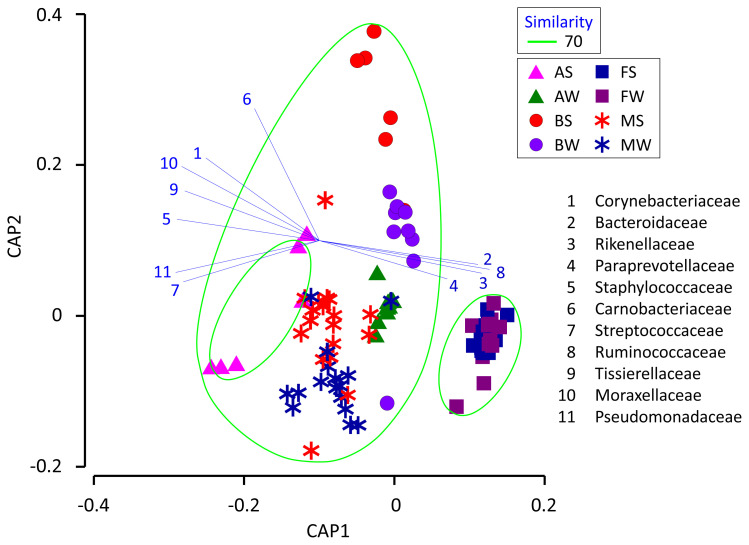
Canonical analysis of principal coordinates plot characterizing the milk, fecal, bedding, and airborne dust microbiota of the dairy farm. AS, AW, BS, BW, FS, FW, MS, and MW indicate airborne dust during summer, airborne dust during winter, bedding during summer, bedding during winter, feces during summer, feces during winter, milk during summer, and milk during winter, respectively. Operational taxonomy units with Pearson’s correlations of >0.7 are overlaid on the plot as vectors. The samples enclosed by the green lines are considered to be in the same group (similarity level 70%).

We used CAP to determine if the milk microbiota was related to the cowshed microbiota (Figure 4). The results indicate that the milk microbiota was grouped together with the bedding and airborne dust microbiota, and not with the fecal microbiota. Likewise, several airborne dust samples taken during summer were low in Ruminococcaceae, Bacteroidaceae, and Lachnospiraceae, and formed a separate group from the others.

## DISCUSSION

Milk yield and protein content follow a seasonal pattern over the course of the year [13]. They are typically greatest during winter and reach a nadir during summer; thus, the higher protein content of the milk obtained during winter was regarded as normal. The dairy cows investigated in the present study were given the same diet (total mixed ration silage) throughout the year, except during the summer when their diet was supplemented with a small amount of palmitic acid calcium. It is therefore difficult to explain why the relative abundances of S24-7, Mogibacteriaceae, and Methanobacteriaceae were greater, and those of Porphyromonadaceae, RF16, and Spirochaetaceae were lower during summer in the fecal microbiota.

Regardless of whether the cows had normal or high SCC, Metzger et al [8] found variation of the milk microbiota between the sampling times after calving, i.e., 1 week, 2 weeks, and 2, 3, 4, or 5 months of lactation. In the present study, only Porphyromonadaceae (decrease), Turicibacteraceae (increase), and Tissierellaceae (increase) exhibited changes in abundance at 1 to 2 months postpartum. This differed from the finding of Metzger et al [8], wherein the abundancies of numerous bacterial taxa varied between sampling times.

Metzger et al [8] also demonstrated that variation of the milk microbiota was greater between seasons than between the sampling times after calving; the abundancies of a large number of bacterial taxa including *Staphylococcus* spp., *Acinetobacter* spp., and *Aerococcus* spp. varied between the seasons. In the present study, only five families exhibited seasonal variation; the abundances of Micrococcaceae, Bacillaceae, and Staphylococcaceae were greater during summer, and Microbacteriaceae and Streptococcaceae were more prevalent during winter. Li et al [9] reported that the abundances of *Pseudomonas* spp., *Propionibacterium* spp., and *Flavobacterium* spp. were negatively correlated with temperature, and those of *Bacillus* spp., *Lactobacillus* spp., and *Bifidobacterium* spp. were positively correlated with temperature. In the present study, we observed a similar temperature effect to that described by Li et al [9] for Bacillaceae (summer > winter).

Regarding the relationships between the milk and cowshed microbiota, the pattern of seasonal variation was the same between the milk and bedding samples for Micrococcaceae (summer > winter), and between the milk and airborne dust samples for Staphylococcaceae and Micrococcaceae (summer > winter). The pattern was reversed between the milk and airborne dust samples for Streptococcaceae. Regarding the relationships between the milk and fecal microbiota, the patterns of seasonal variation (summer > winter or summer < winter) was not the same for any families.

The finding that the milk microbiota was related to the bedding and airborne dust microbiota agreed with the results reported by Wu et al [10]. Although the typical bacterial taxa of feces, i.e., Ruminococcaceae, Bacteroidaceae, Lachnospiraceae, Clostridiaceae, and Rikenellaceae, were stably detected in the milk microbiota, Aerococcaceae, Staphylococcaceae, Moraxellaceae, Corynebacteriaceae, Streptococcaceae, Pseudomonadaceae, and Tissierellaceae, which are regarded as typical bacterial taxa of bedding and airborne dust, were not abundant in the fecal microbiota. Interestingly, Lachnospiraceae and Aerococcaceae were not included in the discriminant vectors with a Pearson correlation >0.7 in the CAP. Instead, Carnobacteriaceae contributed to the differentiated groups. Although the relative abundance of Carnobacteriaceae was <1.0% in all the milk samples, the pattern of seasonal variation (summer > winter) was the same in the milk and bedding samples. Carnobacteriaceae is known as a spoilage-associated taxon in meat [14], although its significance regarding milk quality has yet to be elucidated.

Even though the seasonal variation of the bedding and airborne dust microbiota did not influence the milk microbiota, a distinctive group comprising the milk, bedding, and airborne dust microbiota was formed by the CAP. Therefore, seasonal variation of the milk microbiota mat result from factors that have greater influences than the seasonal variation of the cowshed microbiota. Further research is required to clarify the factors involved in the seasonal variation of the milk microbiota.

Although the importance of the cowshed microbiota has long been recognized, few researchers have investigated the airborne dust microbiota of a dairy farm. Dutkiewicz et al [15] examined cowshed microbiota by plate culture, and isolated numerous species including those belonging to the following genera: *Micrococcus*, *Arthrobacter*, *Staphylococcus*, *Bacillus*, *Corynebacterium*, *Microbacterium*, *Streptomyces*, *Acinetobacter*, *Proteus*, *Pantoea*, *Pseudomonas*, *Thermoactinomyces*, and *Saccharopolyspora*. Although most of the corresponding families, i.e., Micrococcaceae, Staphylococcaceae, Bacillaceae, Corynebacteriaceae, Microbacteriaceae, Moraxellaceae, Enterobacteriaceae, and Pseudomonadaceae, were detected by amplicon sequencing, the abundance of Streptomycetaceae was quite low (<0.01%), and we did not detect Thermoactinomycetaceae in the present study.

The distinctive seasonal variation in the bedding and airborne dust microbiota is difficult to explain, because few relevant surveys have been performed. The dairy farm used fans with a mist of water to cool the bodies of the cows during summer; hence, this enforced ventilation may have caused the differences in the bedding and airborne dust microbiota between the two seasons. The lower abundances during summer than during winter of the five typical bacterial taxa of feces, i.e., Ruminococcaceae, Bacteroidaceae, Lachnospiraceae, Clostridiaceae, and Rikenellaceae, may have resulted from the water mist ventilation.

## Figures and Tables

**Table 1:** Composition of total mixed ration silage produced during summer and winter

Ingredients (% on a wet basis)	Summer	Winter
Whole crop corn silage	27.6	22.9
Whole crop rice silage	4.60	9.15
Timothy hay	3.45	5.26
Alfalfa hay	5.75	3.89
Sudangrass hay	1.84	3.20
Oat hay	1.15	-
Rolled corn	9.84	9.84
Corn gluten meal	1.38	1.38
Corn steep liquor	3.43	3.43
Soybean meal	1.37	1.37
Soy sauce cake	4.14	3.66
Cotton seed meal	0.69	0.69
Beet pulp	5.98	5.95
Molasses	1.26	1.26
Dicalcium phosphate	0.80	0.80
Calcium carbonate	0.92	0.92
Water	25.8	26.3

The total mixed ration mixture was stored after vacuum-sealing in a thick (0.1 mm) plastic bag for 1 to 2 months.

**Table 2:** Milk yield, milk composition, and relative abundance of milk microbiota of the dairy cows examined at one and two months postpartum during the two seasons

Items	Summer		Winter		SEM	p-value	
		
	1M (n = 9)	2M (n = 9)	1M (n = 8)	2M (n = 8)		Season	Month
Milk yield (kg/d)	39.0	39.2	34.6	38.2	2.89	0.449	0.843
Milk composition							
Protein (%)	2.69	2.74	2.94	3.03	0.07	0.003	0.377
Fat (%)	3.48	3.27	3.61	3.56	0.17	0.428	0.304
Solids-not-fat (%)	8.29	8.37	8.45	8.63	0.11	0.182	0.397
Somatic cell count (×10^3^ cells/mL)	74.1	429	65.1	65.6	173	0.035	0.377
Milk microbiota							
Actinobacteria	6.60	11.1	7.62	8.97	1.18	0.017	0.918
Corynebacteriaceae	3.17	5.39	2.84	3.22	0.60	0.168	0.134
Microbacteriaceae	0.25	0.32	0.60	1.02	0.12	<0.001	0.294
Micrococcaceae	0.61	1.32	0.47	0.56	0.28	0.030	0.117
Bifidobacteriaceae	1.28	2.07	1.95	2.44	0.43	0.408	0.278
Bacteroidetes	12.1	9.57	12.8	13.0	1.16	0.221	0.067
Bacteroidaceae	3.18	2.66	3.05	3.14	0.34	0.490	0.418
Porphyromonadaceae	1.53	0.99	1.83	1.36	0.23	0.121	0.048
Rikenellaceae	0.90	0.86	1.13	0.99	0.15	0.214	0.380
S24-7	1.96	0.59	2.31	1.55	0.66	1.000	0.071
Firmicutes	64.9	61.2	61.3	60.2	2.67	0.418	0.490
Bacillaceae	5.63	2.18	0.28	0.47	1.17	0.021	0.770
Staphylococcaceae	9.47	11.2	7.50	7.51	1.93	0.049	0.480
Aerococcaceae	5.34	9.96	4.92	6.07	1.94	0.058	0.071
Lactobacillaceae	3.13	3.82	8.12	4.86	1.36	0.067	0.384
Streptococcaceae	1.58	1.59	2.86	2.51	0.37	0.006	0.438
Turicibacteraceae	1.79	0.48	2.70	1.80	0.64	0.178	0.006
Clostridiaceae	2.06	1.61	1.54	1.74	0.24	0.629	0.796
Lachnospiraceae	5.91	4.84	4.90	5.66	0.59	0.809	0.692
Peptostreptococcaceae	1.41	1.06	0.76	1.13	0.22	0.227	0.593
Ruminococcaceae	11.1	8.30	9.72	10.8	1.15	0.334	0.480
Mogibacteriaceae	1.08	0.86	0.98	0.87	0.12	0.448	0.326
Tissierellaceae	0.86	1.58	1.16	1.59	0.22	0.535	0.008
Erysipelotrichaceae	1.66	0.98	2.07	1.39	0.50	0.535	0.071
Proteobacteria	10.7	13.8	13.5	12.6	1.77	0.361	1.000
Enterobacteriaceae	0.99	1.86	1.76	2.82	0.49	0.067	0.052
Moraxellaceae	3.51	4.30	4.18	3.46	0.58	0.918	0.877
Pseudomonadaceae	1.87	2.65	2.81	2.30	0.46	0.704	0.278

Phyla and families having a relative abundance of >1% in at least one sample are indicated. Summer and winter stand for the sampling conducted between 6 June and 22 August and between 17 November and 2 March, respectively. 1M and 2M indicate one and two months postpartum, respectively.
